# Characterization of Post–exertional Malaise in Patients With Myalgic Encephalomyelitis/Chronic Fatigue Syndrome

**DOI:** 10.3389/fneur.2020.01025

**Published:** 2020-09-18

**Authors:** Barbara Stussman, Ashley Williams, Joseph Snow, Angelique Gavin, Remle Scott, Avindra Nath, Brian Walitt

**Affiliations:** ^1^National Center for Complementary and Integrative Health (NCCIH), National Institutes of Health (NIH), Bethesda, MD, United States; ^2^Oakland University William Beaumont School of Medicine, Rochester, MI, United States; ^3^National Institute of Mental Health, National Institutes of Health (NIH), Bethesda, MD, United States; ^4^National Institute of Neurological Disorders and Stroke (NINDS), National Institutes of Health (NIH), Bethesda, MD, United States; ^5^National Institute of Nursing Research (NINR), National Institutes of Health (NIH), Bethesda, MD, United States

**Keywords:** myalgic encephalitis, chronic fatgue syndrome, post-exertional malaise, exhaustion, cardiopulmonary exercise testing, exercise intolerance

## Abstract

**Background:** Myalgic encephalomyelitis/chronic fatigue syndrome is characterized by persistent and disabling fatigue, exercise intolerance, cognitive difficulty, and musculoskeletal/joint pain. Post–exertional malaise is a worsening of these symptoms after a physical or mental exertion and is considered a central feature of the illness. Scant observations in the available literature provide qualitative assessments of post–exertional malaise in patients with myalgic encephalomyelitis/chronic fatigue syndrome. To enhance our understanding, a series of outpatient focus groups were convened.

**Methods:** Nine focus groups totaling 43 patients who reported being diagnosed with myalgic encephalomyelitis/chronic fatigue syndrome were held between November 2016 and August 2019. Focus groups queried post–exertional malaise in daily life and participants' retrospective memory of post–exertional malaise that followed an exercise provocation with a cardiopulmonary exercise test. Data analysis followed the grounded theory method to systematically code and categorize the data to find meaningful patterns. A qualitative software package was used to move text into categories during data coding.

**Results:** A wide range of symptoms were attributed to exertion both in daily lives and following cardiopulmonary exercise testing. While three core symptoms emerged (exhaustion, cognitive difficulties, and neuromuscular complaints), participants' descriptions were notable for their unique individual variations. Of 18 participants who responded to questions centered around symptoms following a cardiopulmonary exercise test, 17 reported that symptoms started within 24 h and peaked in severity within 72 h following the cardiopulmonary exercise test. Patients described post–exertional malaise as interfering with their ability to lead a “normal” life.

**Conclusion:** The experience of post–exertional malaise in myalgic encephalomyelitis/chronic fatigue syndrome varies greatly between individuals and leads to a diminished quality of life. myalgic encephalomyelitis/chronic fatigue syndrome patients describe post–exertional malaise as all-encompassing with symptoms affecting every part of the body, difficult to predict or manage, and requiring complete bedrest to fully or partially recover. Given the extensive variability in patients, further research identifying subtypes of post–exertional malaise could lead to better targeted therapeutic options.

## Introduction

Persistent and disabling fatigue, exercise intolerance, cognitive difficulties, and musculoskeletal/joint pain are characteristic of a disorder that has been referred to as the Royal Free Disease, benign myalgic encephalomyelitis, chronic fatigue syndrome (CFS), myalgic encephalomyelitis (ME), and systemic exertion intolerance disease at various times in history (1). The term *myalgic encephalomyelitis/chronic fatigue syndrome* is currently the most common term used in diagnostic criteria, by advocates, and by the US Federal Government to refer to the illness (2). Post–exertional malaise (PEM) is a worsening of these symptoms after minimal physical or mental exertion (3). PEM is considered a central feature of ME/CFS (4). The cause of ME/CFS remains unknown, and there are no approved diagnostic tests or treatments (5). Historically, measurement of PEM has had considerable controversy, and patient groups have felt left out of the process by which policy makers develop definitions of ME/CFS (6, 7). Qualitative research affords patients an opportunity to discuss their experiences with researchers at length and inform patient-focused clinical decision making (8).

Previous qualitative assessments of ME/CFS have shown a significant and debilitating effect on the lives of patients. ME/CFS patients have described the fatigue experience as all-encompassing and debilitating, fluctuating, unpredictable, often triggered by minor activities, and causing a significant impediment to daily functioning (7). A previous study using qualitative telephone interviews found the lessening ability to independently perform daily tasks had a significant impact on psychological well-being of ME/CFS patients (9). The one study of which we are aware that employed focus groups to explore PEM in individuals with ME/CFS (10) queried patients about different dimensions of fatigue and found distinct physical and cognitive dimensions, including five key themes: exhausted, drained of energy, heavy feeling, cognitive fog, and muscle weakness.

Previous quantitative studies looking at PEM in ME/CFS patients have shown a wide range of physical and cognitive symptoms affecting every part of the body (6, 11–13). These studies have detailed common PEM symptoms (e.g., physical fatigue, cognitive exhaustion, muscle pain, unrefreshed sleep, and headaches) and timeframes for symptom onset and duration. A recent study (11) used survey data to summarize symptoms, triggers, and time patterns for onset and duration of PEM and found most patients experienced several cognitive and physical symptoms and that triggers of PEM can be physical, cognitive, and emotional. That study also found that onset relative to exertion ranged from immediate to more than 24 h, and duration ranged from <1 h to years.

Although the scientific literature provides few qualitative assessments of PEM in ME/CFS patients, qualitative descriptions of chronic fatigue have been made in other health disorders. A metasynthesis of fatigue across several long-term conditions such as cancer and stroke, not including ME/CFS, produced some commonalities in the fatigue experience (14). For instance, participants described fatigue as unpredictable in occurrence, intensity, and duration and feeling a loss of control of the body. Patients with whiplash-associated disorders suffer from fatigue, sleep disturbance, and cognitive deficits similar to ME/CFS patients (15). Chronic fatigue can be unpredictable and triggered by anxiety and emotional trauma (16). Using the DePaul PEM Questionnaire, one study found the fatigue experience in a subset of cancer patients was similar to PEM in ME/CFS patients (17). However, unlike ME/CFS patients, many patients with fatiguing conditions such as multiple sclerosis and post-polio syndrome are able to exercise without experiencing PEM (13).

Because PEM in ME/CFS patients is still underexplored, especially through purely qualitative methods, the aim of the current study was to expand the knowledge base on the symptoms; manner of onset; timeframes for onset, peak, and duration of PEM; and impact on day-to-day lives of patients. We aimed to understand how ME/CFS is impacted by exertion in day-to-day life and how this compares to the impact after cardiopulmonary exercise testing (CPET). Additionally, we wanted to delve deeper into the experience of PEM following CPET, a gap in the current research. Based on the literature we expected to find a feeling of loss of control and unpredictability to PEM. We also expected to find physical, cognitive, and emotional aspects.

We present results from nine focus groups conducted to better understand PEM experiences from the perspective of ME/CFS patients. Focus groups were centered on ME/CFS patients' usual daily symptoms and how these changed or worsened following exertion. Additionally, for five of the nine focus groups, we recruited ME/CFS patients who had undergone a CPET evaluation and prompted them to report their memory of the symptoms following the CPET evaluation. The primary purpose of the focus groups was to provide a richer and more nuanced understanding of how ME/CFS patients experience their illness. Secondarily, results from the current study were used to inform the design of an exploratory ME/CFS study at the National Institutes of Health (18). We present these findings to aid physicians who provide care to these patients and other investigators interested in designing mechanistic studies of PEM.

## Materials and Methods

We chose to conduct a qualitative assessment through the collection and analyses of rich textual data enabling depth and nuance of discovery not possible via purely quantitative methods. For example, while surveys may produce a comprehensive list of symptoms, focus groups can capture the personal experiences and importance of specific symptoms from the perspective of individual ME/CFS patients. A qualitative exploratory focus group approach was chosen because of its known benefit for underexplored topic areas and disabled populations (19, 20, 23). Focus groups offers the ability to understand the unique experiences of patients and provide a deep, more nuanced understanding of the PEM experience within their social worlds. Focus groups have the further benefit of allowing participants to compare and contrast their experiences, which is particularly helpful when exploring a relatively unknown area. Focus group participants were queried about PEM in their daily lives and in relation to previous CPET tests in which they had participated. Nine focus groups were conducted between November 2016 and August 2019. They ranged from 4 to 7 participants per group for a total of 43 participants and ranged in length from 103 to 120 min. All focus groups were conducted over the telephone to enable geographic diversity without travel burden for the ME/CFS participants.

Data analysis followed the grounded theory method first developed by Glaser and Strauss (21). This approach was chosen to generate a theoretical understanding of the experience of PEM within the social context of persons with ME/CFS. Grounded theory is an inductive, iterative method of conducting qualitative research in which theory is built from the data. Focus group scripts were iteratively modified to further explore emergent categories identified during data analysis. Consistent with the grounded theory approach, data were analyzed using the constant comparative method (22). The constant comparative method is the process of generating conceptual categories from uncategorized data. This involves comparing each piece of data so that similar pieces of data are labeled and grouped to form categories. Every new piece of data is then compared to this categorical structure, and the structure is reconstructed in an iterative manner until no new piece of data challenges the structure's ability to account for all pieces of data (22).

### Participants

Participants were recruited using purposeful sampling, a qualitative sampling procedure in which investigators intentionally recruit participants who have experienced the phenomenon being explored (23). Specifically, 257 potential participants were interviewed by members of the study team. Recruitment solicitations were posted on ME/CFS advocacy websites and were emailed to persons willing to be contacted for research from the practices of health professionals specializing in the evaluation of ME/CFS patients. The majority of these participants were ME/CFS patients in the community and referred by physicians to exercise physiologists for clinical CPET evaluations. All focus group participants reported having received an ME/CFS diagnosis by a health care provider; an independent verification of medical records was not performed.

Within the pool of individuals who expressed interest in participating, we sought to maximize variability with respect to age, gender, race, ethnicity, marital status, education, employment status, severity of impairment (in bed most of the time or not), years since symptoms onset, and geographic location to gain a wide representation. The study was approved by the Combined NeuroScience Institutional Review Board. Informed consent was obtained from all participants using a witnessed telephone consenting process.

### Cardiopulmonary Exercise Testing

CPET is an exercise physiology protocol that is typically used to measure exercise performance and tolerance. It typically involves performing exercise on a cycle ergometer that starts off being easy and steadily becomes harder over time. Participants are instructed to exercise until they reach subjective exhaustion and cannot continue to exercise further (24). Small studies report that a single CPET session (1-day CPET) is a reliable way to induce PEM in ME/CFS patients (25). Single-session CPET is being used as a method to induce PEM for scientific inquiry (26). Some ME/CFS patients undergo an exercise protocol that has them perform two CPET evaluations on sequential days (2-days CPET) as an evaluation of ME/CFS status (27).

As we were interested in learning more about PEM following CPET, five of the nine focus groups were restricted to ME/CFS patients who had undergone CPET to probe them about their experiences with PEM following the test. Of the 18 participants who reported on the timeframe for PEM following CPET, half underwent the 2-days CPET, and half underwent the 1-day CPET. Participants who underwent 2-days CPETs were asked to describe symptoms following Day 1, while also explaining any compounding effects from Day 2.

### Data Collection

All focus groups were conducted by an experienced focus group moderator who had no prior experience with the study population or ME/CFS to ensure impartiality. The semistructured focus group script included broad questions aimed to explore patients' experiences of having PEM, both in their daily lives and in response to the CPET test. Discussion questions centered around activities that can trigger PEM, specific symptoms of PEM, how long after exertion symptoms began, how long the symptoms lasted, and at what point the symptoms were at their worst. Participants were also asked about strategies they employed to try to alleviate symptoms of PEM. With respect to the discussion questions related to the CPET test, we sought to gain a complete picture of how patients felt before the test, during the test, and following the test, including a better understanding of the experience of the onset and course of symptoms. Table 1 shows the final version of discussion questions.

**Table 1:** Focus group discussion questions.

**Focus group discussion questionsDaily post–exertional malaise discussion questions:** • We are interested in learning about how you have felt after exertion in your day to day life. We want to hear about any physical, cognitive, or emotional symptoms that you may have experienced after exertion. It may be helpful to use a specific example. • Probe: Physical, cognitive, and emotional • Probe: We are interested in how you felt throughout your whole body • How long after the exertion in your daily life do your symptoms usually first begin or start to come on? • Please describe the transition from before exertion to having PEM symptoms. • Probe: Was it more gradual or more sudden? • When are your symptoms at the worst, or the peak of PEM following exertion in your daily life? • Probe: How many hours or days after the exertion? • We would like to get a sense of how long after exertion in your daily life until you felt that you had recovered, that is, went back to feeling the same as you did at your usual baseline?
**Cardiopulmonary exercise test discussion questions:** • We are interested in learning about your experiences before, during, and after the CPET test. We want to get a sense of how you were feeling *before* you got on the bike or treadmill, how you felt *while on* the bike or treadmill, and *finally* what you experienced several hours and days later. Please describe what this was like physically, cognitively, and emotionally. • Probe: Physical, cognitive, and emotional. • Probe: We are interested in how you felt throughout your whole body. • How long after the CPET test did your symptoms first begin or start to come on? • Please describe the transition from before the CPET to having PEM symptoms. • Probe: Was it more gradual or more sudden? • When were your symptoms at the worst, or the peak following the CPET test? • Probe: How many hours or days after the CPET? • We would like to get a sense of how long it took after the CPET test until you felt that you had recovered from the test, that is, went back to feeling the same as you did before the test, or at your usual baseline? • Probe: How many hours or days after the CPET? • Please compare the physical, cognitive, and emotional symptoms following CPET with those that occur after exertion in your daily life. In what ways are symptoms after CPET similar or different from symptoms after exertion in your daily life? • Can you describe how it felt as you recovered from the C-PET test? Was it a gradual or more sudden recovery?
**General questions about post–exertional malaise:** • The next question is about any strategies you may have tried to feel better after experiencing symptoms of post–exertional malaise or PEM. Please tell us about anything that you've tried that has or has not helped. • Probe: What does “complete rest” mean? Do you get up for the toilet or to eat? For any other reason? • Have you modified your activities because of anticipation of feeling poorly after exertion? • Probe: What kinds of thoughts go through your mind when deciding whether to exert yourself? • Anything else you would like to add to help us better understand your experiences with PEM related to exertion in your daily life or from the C-PET test? • Any final thoughts or questions before we end today?

At the start of each focus group, participants were given information about the purpose of the discussion and basic ground rules for the discussion such as giving everyone a chance to speak and that there were no right or wrong answers. As is usual during the conduct of focus groups, some participated more than others. However, the moderator systematically solicited participation from each participant and intervened when the discussion veered off topic. Participants often “fed off” each other generating broad and comprehensive discussions. Based on the potential for overexertion, focus groups were limited to 2 h, which allowed for most participants to answer every question; occasionally, not every participant responded to all discussion questions.

### Data Analysis

For reasons explained above, we chose the grounded theory approach and, within that approach, the constant comparative method to analyze our data. Data analysis began after the first focus group and continued iteratively throughout the study. Three researchers developed the coding scheme individually and through team meetings and discussions. In-depth meetings were held to discuss coding differences at length and reach consensus. By the completion of the ninth focus group, salient themes were confirmed and repeated with no new themes emerging (i.e., saturation), signaling an end for the need for further participant recruitment (28). A qualitative software package (29) was used that automated the analysis process described above by allowing the researchers to electronically highlight words or phrases from each transcript and drag them into folders labeled for each theme and subtheme. The software package also allowed for easily combining or separating categories as needed based on analysis.

## Results

Forty-three participants with ME/CFS participated in nine focus groups. Participant demographics are shown in Table 2. Eight overarching themes emerged with salience to ME/CFS patients' experiences with PEM. Themes included the following: (1) PEM was triggered by three broad categories of events; (2) effects of PEM were impacted by baseline pre-exertional symptoms; (3) PEM had a wide symptom range with few differences between daily PEM and PEM following CPET, with three core symptoms (exhaustion, cognitive difficulties, and neuromuscular complaints); (4) PEM following CPET was more immediate and of longer duration than PEM in daily life; (5) the manner of onset of PEM symptoms varied; (6) complete rest was necessary to gain any relief in PEM symptoms; (7) planning and moderation of energy expenditure were seen as essential to avoiding PEM; and (8) the uncertainty and debility of PEM created despair.

**Table 2:** Demographic characteristics of focus group participants (*n* = 43).

**Characteristic**	**Percent (%)**
**Sex**	
Male	20.9
Female	79.1
**Race**	
White	90.7
Black	4.6
Asian	2.3
Native American	2.3
**Ethnicity**	
Hispanic	9.3
Non-Hispanic	90.7
**Age (years)**	
18–29	2.3
30–39	20.1
40–49	18.6
50–59	37.2
60–69	16.3
≥70	4.7
**Marital status**	
Married	48.8
Divorced	14
Living with a partner	9.3
Never married	11.6
**Unknown**	
Education	16.3
High school	4.7
Some college	2.4
Bachelor's degree	48.8
Graduate degree	27.9
Unknown	16.3
**Employment**	
Full-time	2.3
Part-time	9.3
Disabled	67.4
Retired	4.7
Unknown	16.3
**In bed most of the time**	
Yes	42
No	39.5
Unknown	18.6
**Years since symptom onset**	
<5	25.6
5–9	25.6
10–14	18.6
≥15	30.2
**Area of country**	
West	25.6
Midwest	20.9
South	30.2
East	23.3

### Theme 1. PEM Was Triggered by Three Broad Categories of Events

**Figure 1:**
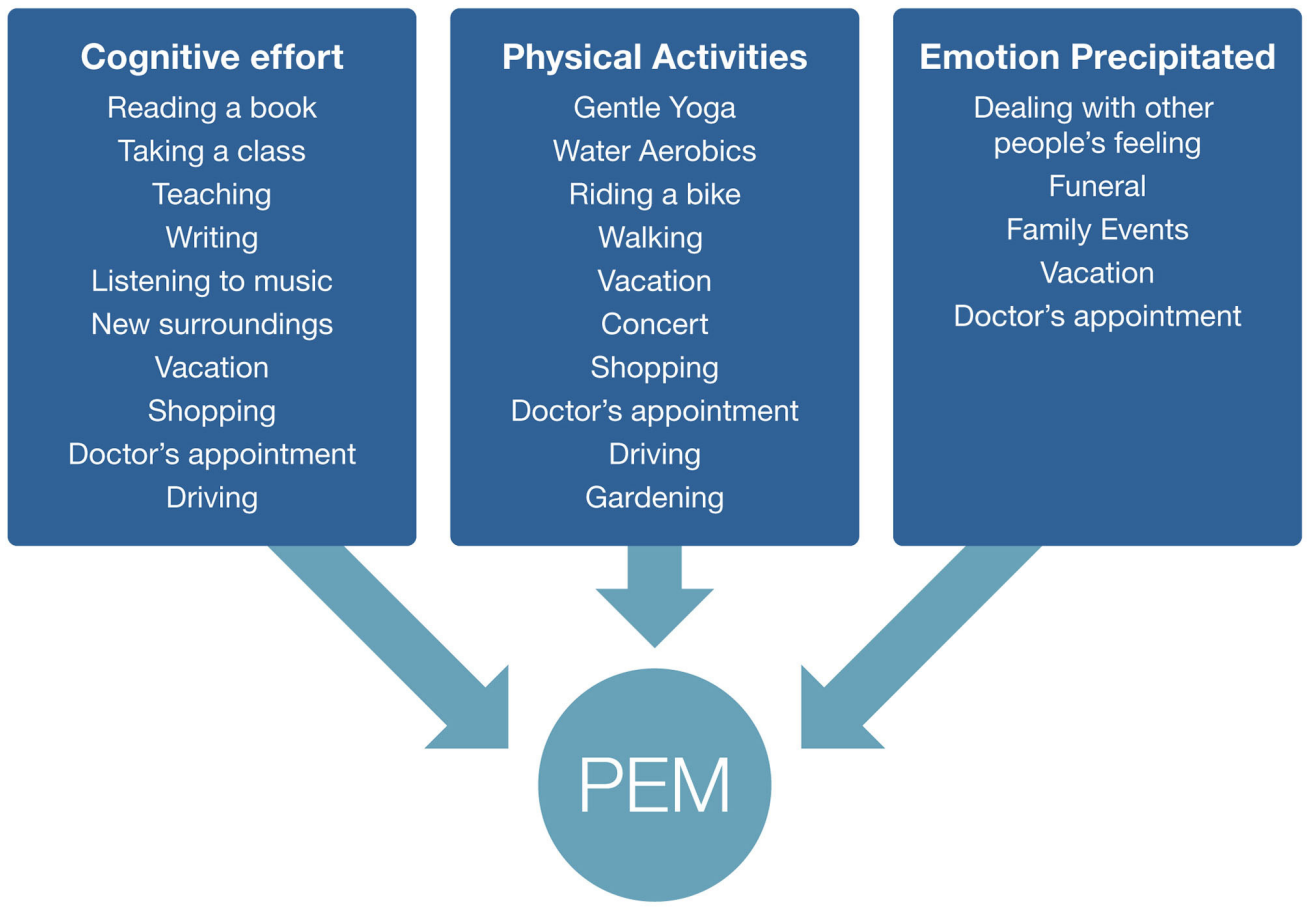
Examples of post–exertional malaise triggers given by focus group respondents. Some triggers fall into multiple categories.

We asked focus group participants to give examples of activities that caused them to have PEM. Notably, there were three broad categories of activities: physical activity, cognitive effort, and emotion precipitated, although there was overlap across the three groups. These categories included triggers such as household chores, social activities, errands outside of the home, physical exercise, cognitive activities, and emotional moments (Figure 1).

One participant explained how a trip to the grocery store can cause PEM:

“*I can go grocery shopping 1 day and I am completely spent for 2 or 3 days.”*

Another participant described how a trip to Walmart can cause PEM:

“*I'm walking through Walmart to get my prescriptions but I'll feel ok, but then as soon as I get home it's like flipping a switch, and I just immediately have no energy.”*

Participants frequently described how cognitive effort can cause PEM symptoms, as this participant described:

“*I specifically notice it if I've had a one-on-one conversation with a friend. After about anywhere from 30 min to an hour, my brain literally starts to shut down, and I can't think clearly and I can't pay attention anymore.”*

Another cognitive trigger example was described:

“*Yesterday I was doing some sorting of a folder trying to clean some things out not even like processing just keeping this, throwing out that, and like an hour of that really burned my brain. I could feel that immediately after.”*

Many participants also described how PEM can be caused by social or emotional stress. One participant described the effect of having her parents visit on a Saturday:

“*Compared to a normal Saturday for me, which is just having my son at home with my husband, I engaged in several hours of social interaction, which I normally don't do. I have all this extra stress of parents coming. It's unexpected and other people in my house and all of that. So the next day midmorning, I start feeling bad and I know I definitely need to rest. So I start feeling bad and I lay down. I am basically in bed for 4 h.”*

Another participant explained how stress can be a trigger for PEM:

“*Stress is a big trigger. If I have a lot of things going on, a lot of things I need to do, or a lot of things I need to accomplish, and/or feel like I need to accomplish, it's hard for me to let go of those things. And I don't get better as quickly if I don't recognize it.”*

### Theme 2. Effects of PEM Were Impacted by Baseline Pre-exertional Symptoms

When focus group participants were asked to describe PEM following exertion, many expressed the importance of understanding their “starting point” or “baseline.” Participants described the pliable nature of symptoms and how successive exertion can compound symptoms. One participant explained this compounding effect:

“*Two days after going to the doctor, my baseline was now exacerbated. It took much less [to cause PEM]. It could now be just having to get in the car and go get my kids, which I do every single day. I'm now unable to do because of that doctor appointment 2 days prior.”*

Having an accurate assessment of baseline was particularly important for PEM following CPET evaluation. Many patients had to travel a long way to get to the site where the CPET was performed. These participants explained that PEM can be compounded by back-to-back exertion and that travel was a trigger for PEM. One participant explained:

“*I had to travel several days across a number of states and I had to fly and all that, so it took a lot out of me just to get to the site of the testing, so I was feeling worse than a typical day for me by the time I got to the test site.”*

Another participant described the effect of the travel to get to the CPET:

“*Flying from Illinois to California and all the traveling, even with having a wheelchair, there was still walking and stress of traveling. I was going in already at a low baseline.”*

Because ME/CFS symptoms can vary widely based on exertion in daily living, to accurately detect changes in symptoms from before to after CPET, it is imperative to obtain a thorough pre-CPET assessment.

This concept was not limited to CPET evaluation, but also was frequently mentioned in relation to daily PEM. Participants emphasized that when they overexert while already in an episode of PEM, the result was amplified.

This participant described how PEM symptoms can compound:

“*If I do make it to the point where I almost faint, it is harder to recover from. Those situations for me are only happening if I just keep letting it compound, if this is several weeks of overexertion or having a cold or another issue.”*

This compounding effect has implications for the management of PEM as described in a later section.

### Theme 3. PEM Had a Wide Symptom Range With Few Differences Between Daily PEM and Following CPET, With Three Core Symptoms (Exhaustion, Cognitive Difficulties, and Neuromuscular Complaints)

**Figure 2:**
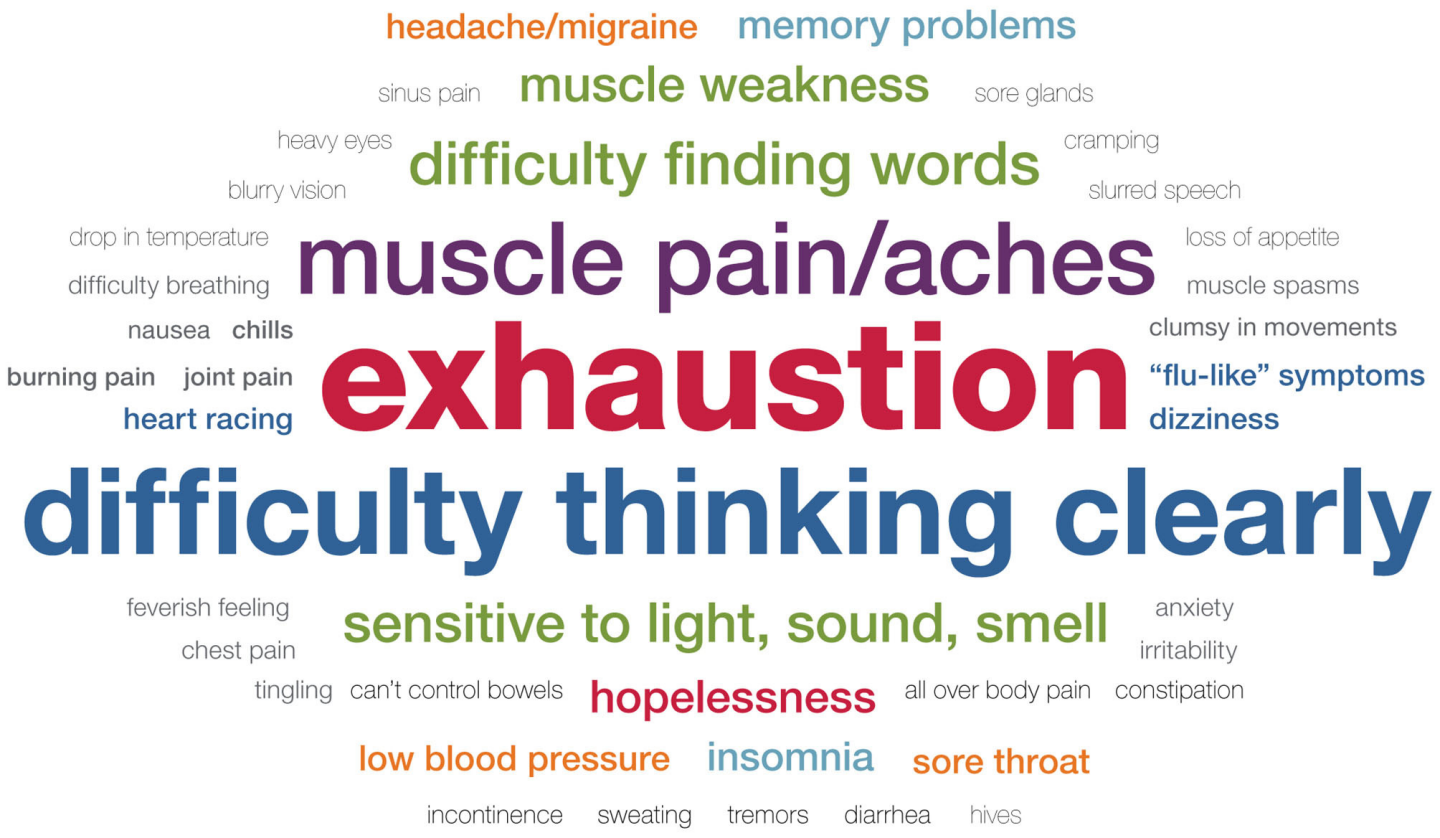
Symptoms of daily post–exertional malaise.

**Figure 3:**
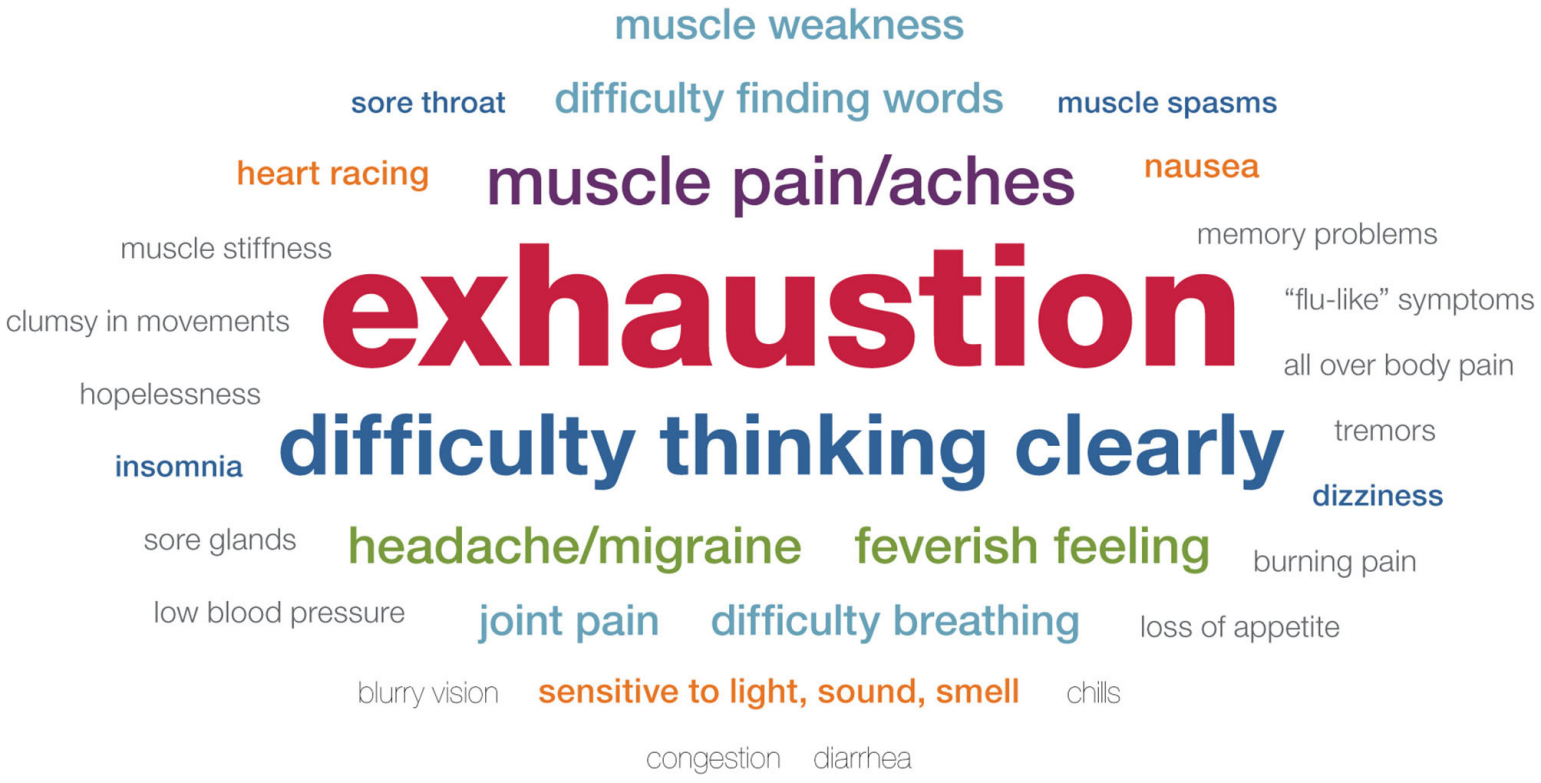
Symptoms of post–exertional malaise following cardiopulmonary exercise test.

During focus groups, we asked participants to describe symptoms they have experienced following exertion, both in their daily lives and following a CPET evaluation. We purposely did not query participants about specific symptoms, but rather asked a general open-ended question, “We'd like to hear some specific examples of how you feel throughout your entire body after exertion.” The purpose of this inquiry was to capture these complaints in the participant's own words. As such, no attempt was made to verify medical symptoms or diagnosis. Additionally, the intention of asking participants about symptoms was to determine the range and most commonly reported symptoms, but it was not feasible in the limited time to query each participant about every potential symptom. Furthermore, the benefit of using focus groups was to capture the most salient symptoms to participants without medical jargon or predetermined categories. Table 3 and Figures 2, 3 present the range and frequency of symptoms reported during focus groups, both for daily PEM and following CPET evaluation. Similar symptoms were reported for daily PEM and PEM following CPET evaluation. In response to this general question, nearly all participants described three core symptoms (exhaustion, cognitive difficulties, and neuromuscular complaints), both for daily PEM and PEM following CPET evaluation.

**Table 3:** Number of focus group participants with self-described post–exertional malaise symptoms.

**Category**	**Symptom**	**Daily PEM (*n* = 30)**	**CPET PEM (*n* = 21)**
**General**			
	Exhaustion	30	20
	Difficulty sleeping/insomnia	8	3
	“Flulike” unspecified	6	2
	Chills	5	1
	Feverish feeling or low-grade fever	3	6
	Drop in temperature	1	—
**Cognitive**			
	Difficulty thinking clearly or paying attention	24	10
	Memory problems	8	2
	Difficulty finding words when speaking	12	5
**Neuromuscular complaints**			
	Muscle pain/aches	20	8
	Muscle weakness	10	5
	Joint pain	5	5
	Muscle stiffness	—	2
	Clumsy in movements	4	2
	Muscle convulsions/twitching/spasms	3	3
	Widespread body pain	2	2
**Sensory**			
	Sensitive to light, sound, smell	11	4
	Blurry vision	—	1
**Affect**			
	Depression/despair/hopelessness	9	2
	Short temper/irritability	3	—
	Anxiety	3	—
**Ear, nose, throat**			
	Sore throat	7	3
	“Sore glands” /lymph nodes	1	2
	Sinus pain	1	—
	Congestion	—	1
	Heavy eyes	1	—
**Cardiovascular**			
	Low blood pressure/near fainting/drop in heart rate	7	2
	Heart racing or pounding	6	4
	Sweating	2	—
**Neurological**			
	Dizziness/vertigo	6	3
	Headache/migraine	7	6
	Burning pain	5	2
	Tingling/numbness	3	—
	Tremors	2	2
	Slurred speech	1	—
	Blurry vision	1	—
**Gastrointestinal**			
	Nausea	4	4
	Diarrhea	2	1
	Can't control bowels	2	—
	Constipation	2	—
	Cramping	1	—
	Loss of appetite	1	2
	Unspecified	2	—
**Pulmonary**			
	Difficulty breathing/short on breath	3	5
	Chest pain	3	—
**Bladder control**		2	—
**Dermatologic**			
	Hives	1	—

*The medical groupings listed on this table are based on the participant's own description of the symptoms. No medical examination was given, nor was there an attempt to verify or qualify self-report symptom*.

#### Theme 3a. Exhaustion

Participants explained that the exhaustion from PEM is different than what they experienced before having ME/CFS. One participant put it this way:

“And it's a flulike exhaustion, really tiring. I used to be an athlete. I had a very intense job. So I would feel a lot of fatigue from those activities. But this is not that type of fatigue. This is a type of fatigue I felt when I rarely got the flu, years ago. Only that flu lasted for a few days and not for the years they have now.”

For some participants, the exhaustion from PEM was severe as explained by a participant:

“On some days, just walking from my bed to the bathroom was exhausting”

Another participant explained how PEM:

“Feels like you've had the flu, and you're just so weak and everything hurts, and you're exhausted trying to take a shower.”

Another participant described exhaustion following CPET as:

“I was exhausted. My arms and legs felt like Jell-O. Like they didn't want to do things.”

Similarly, another explained that after CPET:

“They just had to pick me up and toss me on the bed that they keep next to the bike. I couldn't even get off the bike and onto the bed myself because I was so exhausted.”

#### Theme 3b. Cognitive Difficulties

Cognitive difficulties were described as both difficulty thinking clearly/paying attention and difficulty speaking or finding words.

One participant described cognitive difficulties as:

“I get what I call molasses-type thinking. So I can still think, but it's harder to think and harder to put ideas together. And sometimes I have to read things over a few times to make them stick in my brain.”

Another explained:

“I can't think clearly. I'm unable to make any decisions about anything. Numbers, I feel almost like they're Greek, and they just don't make sense to me anymore at all.”

Thinking clearly was a common complaint as another described:

“I can be in a complete fog for a couple of days, and it is hard to make any decisions or remember basic things.”

Similarly, another participant described:

“I find it much harder to follow a conversation or a story.”

When describing the difficulty talking or finding words, one participant described:

*“With speaking verbally, with words that I have known forever. The words weren't there anymore*.”

#### Theme 3c. Neuromuscular Complaints

Patients often complained of neuromuscular symptoms, which included muscle pain/aches and muscle weakness. One participant described the overall muscle pain as:

“It's like pain has suddenly flared. They'll just be days that are like every exercise in any position I do just hurts. And I try something different, and it hurts and it hurts, and everything is just very irritated and I just have to stop, you know, I can't keep going.”

When describing muscle weakness, a participant talked about a three-block walk:

“I walked three blocks to a CVS and we were in there for maybe 10 min. And I had to leave; my legs were getting so weak they were shaky as if I had just run 10 miles. I had to go out and sit down. We had to go to a coffee place and sit for 20 to 30 min before I could move again to go three blocks back.”

Another participant compared muscle weakness to falling out of a truck:

“Like having glue between my muscles and feeling bruised all over, like I fell out of a truck.”

In addition to the three core symptoms, participants described a wide range of other symptoms including sensory sensitivity, feelings of despair, difficulty sleeping, headaches, nausea, and sore throat, among others (Table 3). Furthermore, no obvious symptom patterns or subsets emerged, but rather PEM symptoms were very specific to the individual. Additionally, patients reported several individual subcategories of major symptoms. For example, within the musculoskeletal subcategory, participants separately described muscle pain and muscle weakness, and as noted above, within cognitive difficulties, participants saw a distinction between difficulty focusing/thinking clearly and difficulty finding words/delayed talking. Participants also told us they view their physical, cognitive, and emotional symptoms as separate. As one participant explained:

“A physical reaction and emotional reaction are just so separate.”

Others explained how the emotional aspects can be tied to the unpredictability of PEM. One participant explained:

“You're questioning what awaits you. It's daily and in every single thing you do, everything you commit to. It's hard.”

While not as commonly mentioned as the three core symptoms, many participants described sensitivity to light, sound, and smell as part of PEM. One participant explained:

“I have to put on earphones. I need to block the sound. I wear a mask to block the light, wear sunglasses.”

When describing sensory sensitivity another participant said:

“I have to keep my room dark and noise to a minimum. If that's not good enough, then I have to wear an eye mask and earplugs to decrease the noise.”

Another talked about her sensitivity to noise:

“Even what's normal noise for people was painful to me. It would make me cry.”

### Theme 4. PEM Following CPET Was More Immediate and of Longer Duration Than PEM in Daily Life

**Figure 4:**
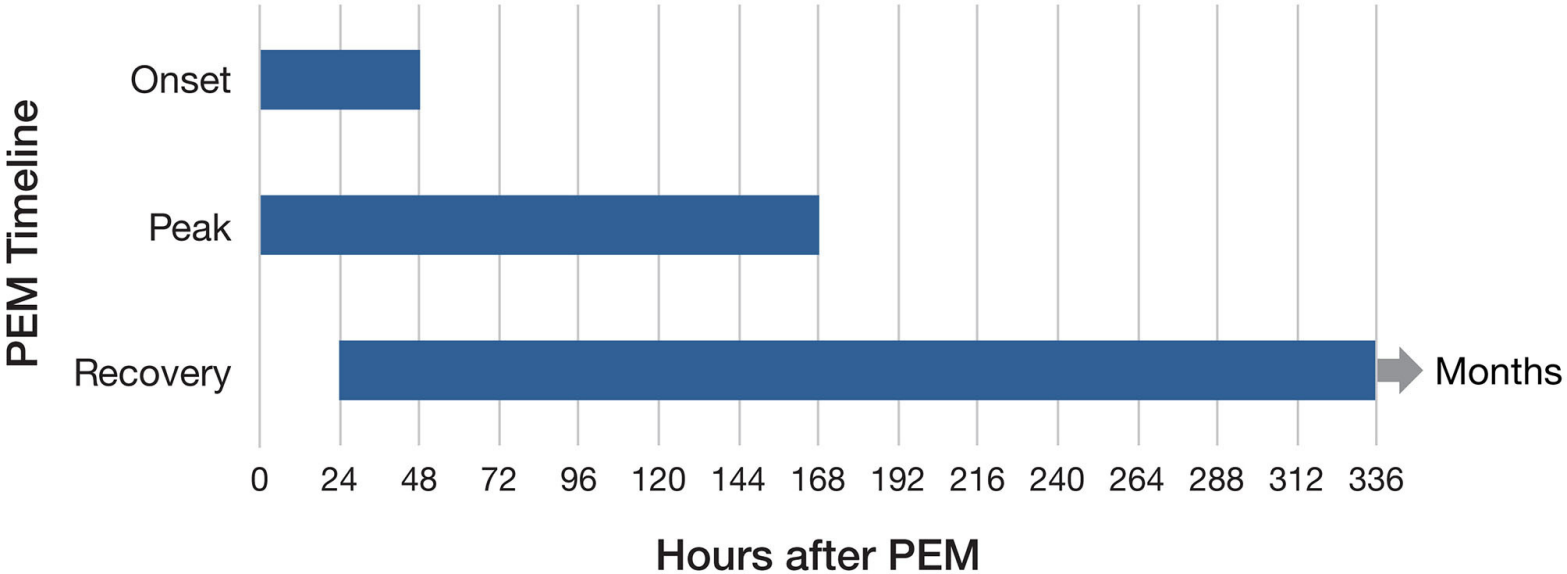
Timeframes for onset, peak, and duration of daily post–exertional malaise.

**Figure 5:**
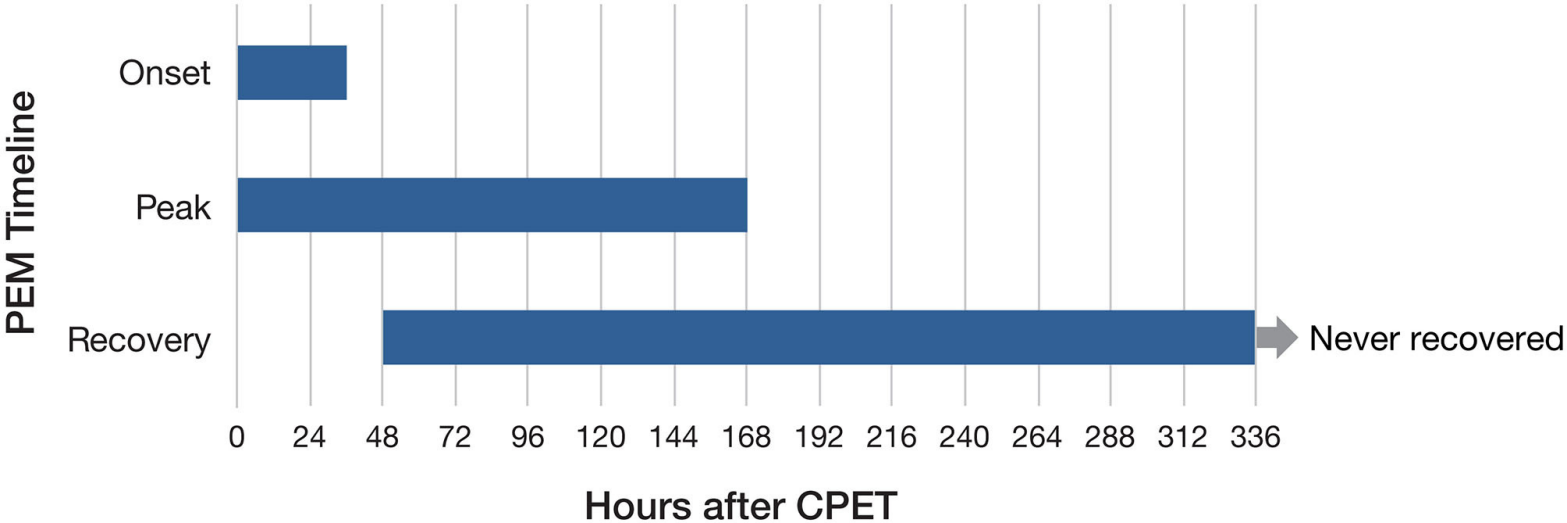
Timeframes for onset, peak, and duration of post–exertional malaise following cardiopulmonary exercise test.

Focus group participants were asked about the timeframes for PEM symptoms, both after exertion in their daily lives and following CPET. We wanted to better understand when PEM symptoms began, peaked, and subsided. Open-ended questions were asked about PEM with no predetermined time frame provided to participants. We analyzed data for post–daily exertion and post-CPET separately (Figures 4, 5). For daily PEM, most participants perceived a delayed onset of symptoms, with nearly half reporting symptoms beginning 12–48 h after exertion. In contrast, more than three-quarters of participants reporting the onset of symptoms following CPET said they began immediately or within several hours. For participants who gave a timeframe for when symptoms of daily PEM peaked, nearly all agreed they peaked within 48 h after exertion, whereas peak in symptoms following CPET was reported sooner, with more than half saying they peaked within 24 h following the test. Approximately half of participants describing PEM from typical daily activities said that symptoms lasted between 2 and 7 days. Half of participants who described PEM symptoms following CPET said the duration was 48–96 h.

Focus group participants contrasted PEM following CPET with PEM in their daily lives. This enabled participants to illustrate how the test pushed them beyond their usual activities. In particular, for many participants PEM following CPET was more immediate than PEM in their daily lives. Fourteen of 18 participants described having symptoms immediately or within a few hours following CPET compared to less than half of participants describing daily PEM. Many participants described sudden and immediate symptoms following the CPET, and for some, these began while still exercising on the bike, as described by this participant:

“During the test and right after I felt terrible. I felt I was going to pass out and very out of breath. I felt extremely nauseous like I was going to throw up. I felt very weak, and I was shaking, and they had to help get me off the bike.”

Another participant explained:

“As soon as I got off of the bike, I was incredibly wobbly. My muscles weren't working right, like I couldn't get them to work well. So they had me lie flat immediately for about an hour. During that time I started to feel sick. And by the time I got back to the hotel, I was in bed for the rest of the day.”

In addition to having more immediate symptoms following CPET, many also talked about how the CPET symptoms were more severe than PEM symptoms in daily life. As one participant told us:

“It was radically different than what normal life is because a lot of energy was expended in a short period of time… My day to day life is much different than that. I don't normally use energy that quickly and in that quantity. It's usually more of a gradual pronounced thing, whether it's working around the house a little bit, doing different chores… Normally that buildup of energy happens over a longer period of time.”

Another explained that:

“The symptoms [following CPET] were similar to PEM in day to day life, but they're multiplied by a factor of five, every one of them.”

Another participant agreed the symptoms were similar but more intense:

“I'm not sure the symptoms were a whole lot different than what I normally experience. It was just so much all at once.”

Reinforcing that PEM following CPET was more severe than daily PEM, this participant described how she still has not fully recovered:

“By the time I got home, I was pretty much a wreck. I was able to walk into the house on my own with my husband helping hold me up but I was unable to function at all. I wasn't brushing my teeth. I was just focused on getting to the bathroom. And I would say it took 4 months before I came back to close to my baseline. I don't think I've ever returned back to what I was before I walked into that test.”

### Theme 5. The Manner of Onset of PEM Symptoms Varied

Separate from when PEM began (as shown in Figures 4, 5), we also determined whether participants perceived the onset of symptoms as sudden or gradual. We asked them to describe the transition from before exertion to experiencing symptoms of PEM. Many participants explained that it varied such that some symptoms came on suddenly and other symptoms were more gradual. As one participant explained:

“The hand tremors were sudden. The other symptoms I would say were more gradual. The other symptoms being the body pain, the diarrhea, the low-grade fever.”

For participants who reported their symptoms to have a usual onset, they were nearly evenly split between gradual and sudden onset. One participant described the gradual onset of symptoms as:

“It was gradual. The symptoms just started coming on, and they just kept getting a little bit worse and a little bit worse and a little bit worse.”

Another participant said that for her the onset is usually sudden. She explained:

“The symptoms often happen with an episode of low blood pressure, near fainting experience. And so, when they're combined, it's very sudden. And I can have an episode of almost fainting that comes on within minutes.”

Interestingly, 11 focus group participants described experiencing an adrenaline rush while doing an activity before the PEM symptoms came on, both in daily activities and during the CPET. These participants described experiencing “adrenaline,” “endorphins,” and “euphoria.” One participant described this feeling after CPET as:

“I get that high of feeling like, wow, I can do anything…”

Another explained:

*“Emotionally right after my test I felt elated*.”

Another said,

*“I had a surge of endorphins and adrenaline during the test*.”

### Theme 6. Complete Rest Was Necessary to Gain Any Relief in PEM Symptoms

When asked what could alleviate PEM symptoms, virtually every participant agreed that while in an episode of PEM, complete rest was absolutely necessary to reduce symptoms. Many participants emphasized that this was not a strategy so much as an outcome. For these participants, complete rest was a “demand from the body.” One participant described it as:

“I have to say that the only thing that helps me, that helps those symptoms start to subside is complete rest. There is not a drug, there is nothing. It is complete rest”

Another participant elaborated,

“Complete rest is really the only thing that can facilitate a recovery for me. Basically, I have to stop and put things on hold because I realize when I am weakened with PEM, if I push through… I will end up making the symptoms worse, like going downhill really fast.”

Another described:

“It feels like life has all but shut down. It's such a profound undercutting of everything that feels positive, everything that feels like you can make a movement out or forward or up. Somebody was talking about the need to lie down. It's not even just a need. It's an absolute necessity. And I think that PEM just courses through your body and steals everything away that you think of as lively.”

When asked to describe what “complete rest” entails, most participants described lying down “absolutely flat” and with as little sensory input as possible. For many, this included ear plugs, darkness, and solitude. Some participants minimized going to the bathroom or had a bedside toilet option. In addition to complete rest, participants described a wide array of practices including over-the-counter and prescription medications, relaxation techniques, special diets, and professional counseling.

### Theme 7. Planning and Moderation of Energy Expenditure Was Essential to Avoiding PEM

An interesting theme that emerged during focus group discussions centered around the steps taken by participants to manage activity levels in their daily lives to minimize the effects of PEM. This evolved into an in-depth discussion of pacing and its importance to ME/CFS patients in coping with the illness. Many participants described months or years of learning strategies for mitigating PEM. One participant explained:

“Now that I know how to manage my illness better and I know to always rest more than I need to, I don't have big crashes very often. I'm able to maintain a certain equilibrium as long as I stay within my energy envelope, and I have to be very strict about it. I've missed weddings and funerals and births and birthdays, and I have to be very, very careful, but I'm doing better overall, and my quality of life from day to day is better. And, so, I don't have very many crashes. So, I've kind of finally figured out how to keep that equilibrium, but it's very tentative.”

Another participant described the importance of planning ahead:

“If it's a really big thing, say I know that I've got to run a lot of errands that are unavoidable, I will actually look at the calendar and make sure that I don't have anything back to back, there's nothing going on for days after.”

Calendar management was an important aspect as a participant described:

“The other part of it is really, really managing my calendar. If I have a doctor's appointment, there is literally nothing else that I can get done that day and I have in my head to be prepared for it. So, I keep lots of tasks lists and things that need to get done, and during the week, I sort of move things around or, you know, change things.”

Along with learning to pace themselves, many participants described the compounding aspect of PEM. One participant explained:

*“If I'm already in PEM and overexert, I feel the effects instantly and more intensely, and it lasts deeper and longer*.”

Another explained:

*“It's not just an add-on it's a multiplier. It's like an exponential effect on it. So to overdo while you're having PEM is much worse than overdoing when you're not in PEM*.”

Although participants talked at length about the importance of moderating activities, many also emphasized that it is not easy, and PEM can be unpredictable. As one participant explained:

“One of the confusing things about symptoms is that they sometimes respond to behavioral changes, so, e.g., not doing certain things in order to not trigger the symptoms. And yet, other times is seems to happen no matter what you try to do differently. It's just not easy to predict or mange.”

Finally, many participants talked about the “learning curve” involved in managing activities in order to avoid PEM. Many took years of overexerting and “crashing” before learning better how to manage having ME/CFS. One participant put it this way:

“When I first got sick, I wasn't even familiar with the concept of pacing. So I was constantly in the cycle of overdoing it and crashing and overdoing it and crashing.”

### Theme 8. The Uncertainty and Debility of PEM Created Despair

We asked focus group participants to describe the emotional aspects of having ME/CFS and PEM in particular. Participants talked at length about living with the unpredictability of PEM and having to adjust their lives to try to avoid severe PEM. Participants described the anxiety of not knowing how long the PEM would last and if they would ever return to their pre-PEM state. One participant summed it up as,

*“I have a kind of post exertional despair that maybe I'll never get better*.”

Other respondents described the difficulty in having ME/CFS symptoms on a daily basis and knowing that PEM could occur at any time, such as a participant who said:

*“The real hard part is that you have to choose. You can't just do this. My life is never going to be complete*.”

Another participant explained the unpredictability of PEM:

“It seems unpredictable in my case because I could do the same thing two different days, and 1 day it affects me a lot more than the other day.”

Another explained the despair in living with PEM:

*“I have been sick so long that I really don't have a life. I would give anything to have just some part of my life back*.”

Another participant explained the toll PEM has taken on her life,

*“It's substantially different than my life was before, and it's debilitating to my life*.”

## Patient's Experiences of PEM

**Figure 6:**
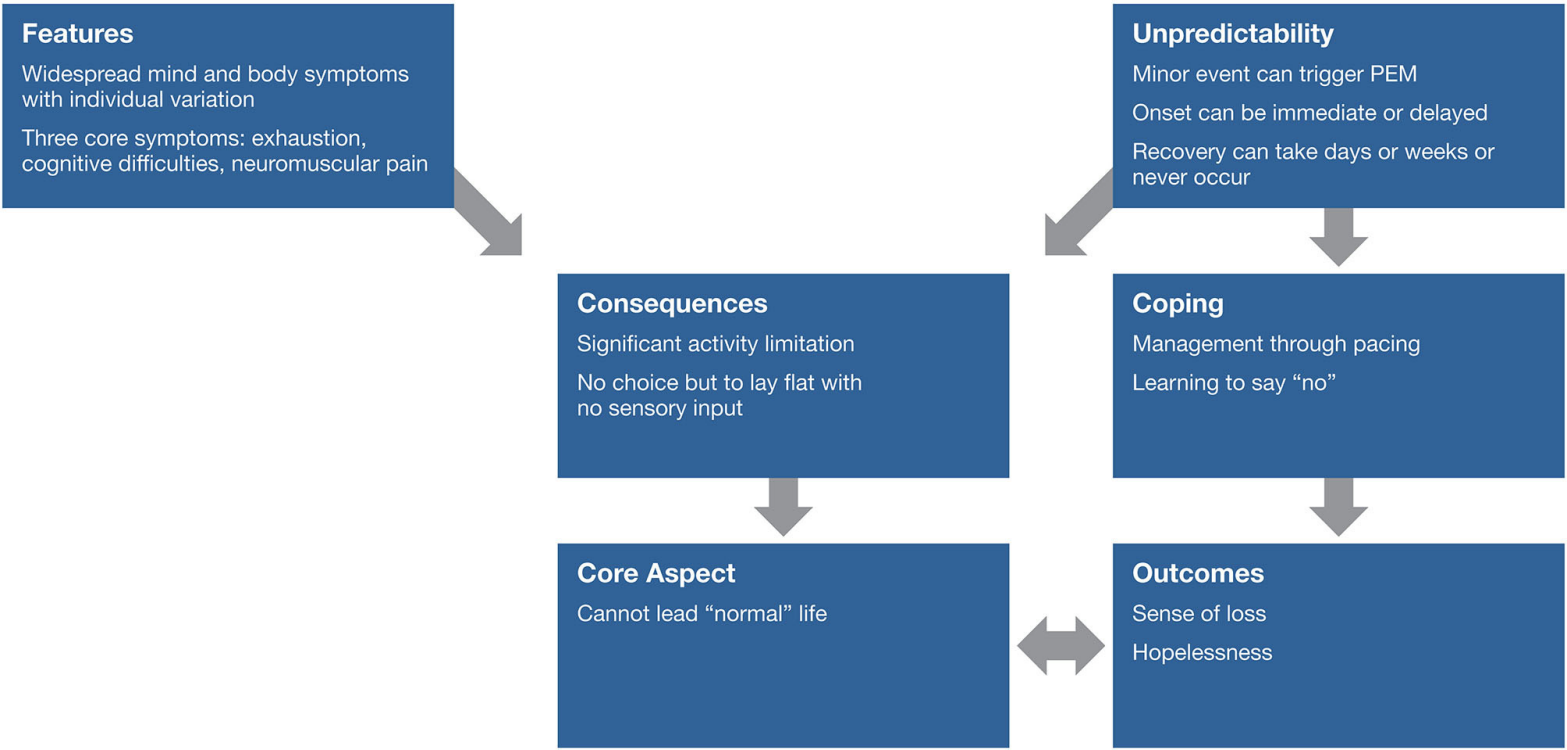
Experience of post–exertional malaise.

Figure 6 diagrams the overall experience of PEM described by focus group participants. Focus group analysis revealed an inability to live a “normal” life as a core aspect of the PEM experience as described by patients. The widespread mind and body symptoms coupled with the unpredictability of triggering events and the timing of onset and recovery of PEM create disabling consequences for ME/CFS patients. While many patients have found some success in managing PEM through pacing and forgoing previously joyful activities, our analyses nevertheless revealed a profound sense of loss and hopelessness in several participants. When describing the symptoms, timeframe, and experience of PEM, many found it helpful to contrast PEM to how “normal” people experience energy. An example is a participant describing her lack of energy throughout the day,

“A normal person's energy is almost energy on demand throughout the day aside from resting every 24 h, but for us, it is such a lag for recuperating energy.”

Another explained:

“I feel like I'm just constantly assessing my energy level, and normal people don't do that. They get up in the morning and they pretty much know that they can get through a list of things to do, whereas it can take me weeks to get two or three things done, sometimes none at all.”

Focus group participants similarly contrasted their cognitive fatigue with “normal” people as this participant explained:

“You cannot focus on simple things like remembering the name of a lamp… the word won't come. I can't balance a checkbook, can't do any kind of math, can't absorb information. People will be explaining something to you, and it's like they're speaking another language, and my mind will not focus on what they're saying. Those are times I stay home because I shouldn't be driving. I shouldn't be operating any kind of machinery. I shouldn't be cooking because I'm not able to function on a ‘normal' basis like everybody else does.”

Participants also described the inability to live a “normal” life due to the compounding effect of PEM. One participant explained this in relation to a visit from relatives,

“So my parents decide to come, and it immediately becomes this stressful situation because my mom is a delegator, and she's saying we need to bring our dog and start looking for a dog-friendly hotel and a place to stay with her RV, and she's already delegating and putting this stuff on me without realizing what she's doing. So all of that may not seem like much to a normal healthy person, but that starts building throughout the day.”

This lack of living a normal life came up related to adjustments people have made in their daily lives to try to minimize PEM. As one participant described:

“There are all sorts of things we do to try to minimize PEM. I used to listen to music and I don't do that anymore. And there are all sorts of things that when anyone sees me doing this, I look pretty normal. But they don't see all the planning and all the changes I've made in order to do something. Like going outside, I used to hike. I can't do that anymore. But I can still sit down and picnic, and that looks normal to someone else.”

## Discussion

Focus groups analyses found that PEM is significantly disruptive to the lives of ME/CFS patients, often being unpredictable and difficult to control. Day-to-day activities such as going to the grocery store or having a family member visit can cause PEM, and symptoms are wide ranging with every part of the body affected. This is the first in the literature using purely qualitative methods to study PEM following CPET, and findings point to more immediate and longer-lasting PEM than occurring in patients' day-to-day lives. Participants also described the necessity of lying flat and minimizing sensory input to recover from PEM and tedious planning to try to avoid episodes of PEM. The PEM experience for ME/CFS patients can create a significant emotional impact.

The wide range of symptoms found in the current study has been found in previous research. For instance, a previous review article found that symptoms affect every part of the body (30). Similarly, Chu et al. (11) found over a dozen PEM symptoms affecting all parts of the body and also noted a different cluster of symptoms among men and older patients vs. women. The current study found no discrete symptom groups, and in fact, many individual symptoms were reported by only one or two participants.

In addition to wide-ranging symptoms, the current study found three core PEM symptoms: exhaustion, cognitive difficulties, and neuromuscular complaints. While several studies have found core PEM symptoms, the exact set differs across studies, although nearly all have found some form of physical fatigue, cognitive difficulties, and pain as core symptoms. A recent study (31) used open-ended questionnaire data and predefined symptom categories to determine PEM symptoms in ME/CFS patients following CPET testing. That study found fatigue, muscle/joint pain, and cognitive dysfunction occurred with greatest frequency, overlapping substantially with the current findings. A previous study examining PEM across several countries found fatigue, cognitive dysfunction, disturbed/unrefreshed sleep, and pain presenting as common symptoms across patients (32). Another study found fatigue, difficulty concentrating, difficulty thinking, and muscle pain as the top four PEM symptoms in ME/CFS patients (11), and another found reduced stamina, physical fatigue, cognitive exhaustion, and problems thinking to be the most common PEM symptoms (6). Several studies have found unrefreshed sleep/sleep disturbance as a core PEM symptom reported via questionnaire data (6, 11). Sleep disturbances or unrefreshed sleep was reported by about one-quarter of participants in the current study. One-third of participants in the current study reported sensory sensitivity, higher in frequency than previous studies using questionnaire data (11, 12). Differences across studies may be due to differences in data collection methods and/or differences in the demographics of the study populations. None used a random sample of patients, and most used an exhaustive list of symptoms rather than the open-ended approach used in the current study.

Our findings overlap substantially with a previous focus group study of PEM in ME/CFS patients. That study found five main themes related to symptoms: feeling exhausted or tired, feeling heaviness in the limbs or whole body, sensing fogginess in the head, feeling weakness in the muscles, and feeling drained of energy (10). These findings overlap with the three core symptoms that emerged from the current study. “Cognitive difficulties” is comparable to “sensing fogginess in the head,” “muscle pain and weakness” is akin to “feeling weakness in the muscles,” and “exhaustion” overlaps with “feeling drained of energy” and “feeling exhausted or tired (11).” This previous study found that physical or cognitive exertion can cause PEM, and basic daily activities such as bathing, dressing, toileting, and reading can be triggers. The current study similarly found a wide range of physical and cognitive activities can trigger PEM, but also found that emotional events are common triggers. Focus group participants described how the emotional stress from family visits or funerals can trigger PEM symptoms. Previous studies using questionnaires have similarly found that emotional distress can cause PEM (6, 11, 33).

The current findings in conjunction with previous studies presenting descriptors of PEM symptoms have translated into multiple labels used for similar symptoms. For example, Chu et al. (11) list “poor concentration” and “difficulty thinking” separately among the top symptoms, whereas Holtzman et al. (6) list “cognitive exhaustion” and “problems thinking” separately. The current study found differences reported by participants between “difficulty focusing or thinking clearly,” “memory problems,” and “delayed speech or difficulty finding words.” These cognitive symptoms may correspond to the neurocognitive domains of attention/executive functioning, memory, and language functioning, although it is also possible that cognitive symptoms that appear to be describing deficits in one domain (e.g., language) are actually the downstream effect of disruption of another domain (e.g., attention/executive functioning). Regardless, in conducting neurocognitive and neuroimaging studies of ME/CFS, the current study may suggest focused assessment of those three cognitive domains and the brain networks that subserve them. The wide variety of ways in which people describe the same experience may cause inconsistencies in the performance of patient outcome questionnaires used to measure PEM symptoms. Underreporting of symptoms with PEM symptom questionnaires has been previously observed (34). Open-ended questions to allow research participants to express their personal nuances of PEM may be needed in addition to standardized instruments, to accurately discern the onset and severity of PEM in an experimental setting.

PEM in the current study was similar to PEM found in veterans with Gulf War syndrome who rated exercise as painful and fatiguing (35). However, prolonged effects from PEM occurred more often and with greater duration among ME/CFS (13) patients than patients with multiple sclerosis and postpolio syndrome, suggesting that the fatigue experience is multifaceted with variation across patient groups.

The current study is the first in the available literature using qualitative methods to compare daily PEM and PEM following CPET evaluation. Patients emphasized the importance of understanding pre-exertion state to fully assess the effects of PEM. Furthermore, current findings highlight that PEM following CPET was more immediate and of longer duration than PEM in daily life. For both daily and following CPET, participants said recovery took several days to several weeks or even months with more variation seen for daily PEM. These findings are in keeping with the literature. Jason et al. (34) found variability in the duration or onset of fatigue after activity, from an hour to over a day. Another ME/CFS study (10) found PEM came on immediately for some, and for others, it was delayed and that it often depended on the intensity of activity. Many of the current participants also described variability based on the intensity of activity and whether they were already in the midst of a PEM episode.

Also unique to the current study is querying patients about whether they perceived a sudden or gradual onset of PEM symptoms. Like other aspects of ME/CFS, no clear pattern was seen. Regardless of specific symptoms, timing, and onset of PEM, participants nearly all agreed that recovery from PEM required complete rest, and this rest must include as little sensory input as possible.

The need on the part of ME/CFS patients for calendar management and pacing has been seen in several prior studies (36–38). A prior focus group study found that patients can benefit by learning their body signals and by individually tailored activities (36). Clinical and experimental studies should consider providing schedules to participants prior to the study to enable alterations to be made to aid in participant pacing. Likewise, every attempt should be made to allow ME/CFS participants to have complete rest to recover from PEM. Studies of PEM should be cognizant of this need in providing after CPET care for ME/CFS participants. Pacing has also been found as beneficial with postpolio syndrome (39) and chronic pain and fatigue (40).

The current study touched upon the emotional toll of coping with ME/CFS. Researchers and clinicians should take care to appreciate and address the deep despair conveyed by ME/CFS patients. For some, the daily toll of living with ME/CFS has been devastating. There currently exist few treatments for ME/CFS and current clinical protocols focus on management. A previous study found that stress management interventions might alleviate PEM in some patients (41). The current finding that emotional triggers can cause PEM adds additional evidence that stress management could be beneficial.

## Study Limitations

There are several limitations to this study. First, responses were dependent on retrospective recall of participant experiences with CPET evaluation. Second, all of the participants provided their diagnostic information strictly through self-report. No attempt to understand whether participants would fulfill a criteria-based diagnosis was made as part of this study. These participants are best described as persons who reported a medical diagnosis of ME/CFS and had a physician refer them for ME/CFS specific CPET testing in the community. The current research team has had extensive experience with review of medical records for ME/CFS; the vast majority of diagnoses are made by practitioner gestalt rather than by published diagnostic criteria. These results reflect how PEM is described by persons in the general ME/CFS community. Third, focus groups were conducted over the telephone, making it more difficult for the moderator to control and manage discussions. Some participants may not have fully engaged with the focus group, and at times, discussions went on tangents unrelated to the original query. Despite these limitations, almost all participants contributed substantially to discussions, and the moderator was able, to a large extent, to keep discussions focused and on track.

## Implications for Future Research and Clinical Application

The current study points to several areas that warrant further exploration. One such area is determining the most effective tools clinicians can provide to patients for managing PEM. Because of the lack of effective treatments for PEM, some ME/CFS researchers have suggested pacing as a therapeutic option to be used by practitioners (42). Focus group participants in the current study talked at length about the importance of planning and moderation of energy expenditure to avoid PEM, and many described a long period of trial and error before gaining any success with moderating PEM. Although widely discussed in patient forums, this topic has little empirical research and should be studied further. In particular, future research could identify specific pacing regimens that prove most beneficial to specific subtypes of PEM.

The current study also points to the need for researchers studying PEM in ME/CFS patients to be cognizant of the effects of travel on PEM. For example, patients should arrive several days prior to starting participation to foster recovery from travel. Additionally, it is important to fully understanding a patient's pre-CPET state to accurately assess the effects of the test on PEM symptoms. ME/CFS patients described a fluid baseline, which could change quickly and was difficult to anticipate. Assessments should be performed before the patient travels to get an accurate understanding of the patient's physical, cognitive, and emotional state prior to the experiment. Researchers should also note that PEM induced by CPET differs from daily PEM, and symptoms and timeframes from the experimental setting might not fully correspond with those found in daily PEM.

## Conclusion

ME/CFS patients describe PEM as all-encompassing with symptoms affecting every part of the body, difficult to predict or manage, and requiring complete bedrest to fully or partially recover. Through in-depth focus group discussions, ME/CFS patients describe PEM as disruptive to living a self-described “normal” life, sometimes leading to hopelessness or despair. Given the extensive variability in PEM symptoms and timeframes for onset, peak, and recovery, further research identifying subtypes of PEM could lead to better targeted therapeutic options.

## Data Availability Statement

The datasets generated for this study will not be made publicly available The data are all textual and cannot be provided without breaching confidentiality.

## Ethics Statement

The studies involving human participants were reviewed and approved by NIH Combined NeuroScience Institutional Review Board. The patients/participants provided their written informed consent to participate in this study.

## Author Contributions

BS designed the study, collected and analyzed the data, wrote, and edited the manuscript. AW, AG, and RS collected and analyzed the data and edited the manuscript. JS participated in writing and editing the paper and approved of the final version. AN contributed to supervision of the work, participated in writing and editing the paper, and approved the final version. BW contributed to supervision of the work, data collection, data analysis, writing and editing the paper, and approved the final version. All authors contributed to the article and approved the submitted version.

## Conflict of Interest

The authors declare that the research was conducted in the absence of any commercial or financial relationships that could be construed as a potential conflict of interest.

## Abbreviations

ME/CFS: myalgic encephalomyelitis/chronic fatigue syndrome
PEM: post–exertional malaise
CPET: cardiopulmonary exercise test.
